# Comparative proteomic profiling of the serum differentiates pancreatic cancer from chronic pancreatitis

**DOI:** 10.1002/cam4.1107

**Published:** 2017-06-01

**Authors:** Mayank Saraswat, Sakari Joenväärä, Hanna Seppänen, Harri Mustonen, Caj Haglund, Risto Renkonen

**Affiliations:** ^1^ Transplantation Laboratory Haartman Institute University of Helsinki Helsinki Finland; ^2^ HUSLAB Helsinki University Hospital Helsinki Finland; ^3^ Department of Surgery University of Helsinki and Helsinki University Hospital Helsinki Finland; ^4^ Translational Cancer Biology Program Research Programs Unit University of Helsinki Helsinki Finland

**Keywords:** Chronic pancreatitis, HDMSE, OPLS‐DA, Pancreatic adenocarcinoma, Pancreatic cancer

## Abstract

Finland ranks sixth among the countries having highest incidence rate of pancreatic cancer with mortality roughly equaling incidence. The average age of diagnosis for pancreatic cancer is 69 years in Nordic males, whereas the average age of diagnosis of chronic pancreatitis is 40–50 years, however, many cases overlap in age. By radiology, the evaluation of a pancreatic mass, that is, the differential diagnosis between chronic pancreatitis and pancreatic cancer is often difficult. Preoperative needle biopsies are difficult to obtain and are demanding to interpret. New blood based biomarkers are needed. The accuracy of the only established biomarker for pancreatic cancer, CA 19‐9 is rather poor in differentiating between benign and malignant mass of the pancreas. In this study, we have performed mass spectrometry analysis (High Definition MS^E^) of serum samples from patients with chronic pancreatitis (13) and pancreatic cancer (22). We have quantified 291 proteins and performed detailed statistical analysis such as principal component analysis, orthogonal partial least square discriminant analysis and receiver operating curve analysis. The proteomic signature of chronic pancreatitis versus pancreatic cancer samples was able to separate the two groups by multiple statistical techniques. Some of the enriched pathways in the proteomic dataset were LXR/RXR activation, complement and coagulation systems and inflammatory response. We propose that multiple high‐confidence biomarker candidates in our pilot study including Inter‐alpha‐trypsin inhibitor heavy chain H2 (Area under the curve, AUC: 0.947), protein AMBP (AUC: 0.951) and prothrombin (AUC: 0.917), which should be further evaluated in larger patient series as potential new biomarkers for differential diagnosis.

## Introduction

The age‐standardized incidence rate for pancreatic cancer (PC) in men has increased by 25% from 1957 to 2011 in Finland (http://www.cancer.fi/syoparekisteri/en/). The average age at diagnosis for PC is 69 years in Nordic males and 72 years in females. The average age at diagnosis for chronic pancreatitis (CP) is lower, 40–50 years, but there is an overlap in age, and the differential diagnosis between CP and PC can be very difficult. Clinical features, imaging changes and macroscopic appearance of CP may be difficult to differentiate from PC and a special challenge may be with paraduodenal and autoimmune pancreatitis because they closely resemble those of pancreatic ductal adenocarcinoma (PDAC).

There is no reliable non‐invasive method to differentiate PC and CP at the moment and invasive methods such as needle biopsies are neither complication‐free nor reliable.

The only established serum marker for pancreatitis is CA 19‐9 with a sensitivity and specificity of 68% and 70% in differentiating between benign and malignant masses in the pancreas at the cutoff of 37 U/mL 1. Increasing the cutoff increases the specificity markedly but sensitivity goes down markedly. New sensitive and specific biomarkers for differentiating these two diseases are desperately needed in the clinic. High throughput plasma proteomics can provide biomarker candidates which differentiate between the two diseases. Previously, some proteomic studies have been conducted to find biomarkers for pancreatic cancer which support and validate current study 2, 3, 4, 5, 6.

We have used high definition MS^E^ (HDMS^E^) methodology to compare chronic pancreatitis and pancreatic cancer in serum samples. We have quantitated 291 proteins including both classes of the samples. All protein abundances were analyzed separately between the PC and CP patients. Data were further analyzed by principal component analysis (PCA) and orthogonal partial least square discriminant analysis was employed to classify the samples and find out the most significantly differing proteins between the patient groups. We propose multiple high‐confidence target biomarkers which are able to classify the two diseases into separate groups.

## Materials and Methods

### Patient samples

Preoperative serum samples according to the routine of the hospital laboratory were collected from 22 patients with pancreatic cancer and 13 patients with chronic pancreatitis. Of the patients with pancreatic cancer, all underwent pancreaticoduodenal resection with curative intent. Of the patients with chronic pancreatitis 10 patients had alcohol‐induced chronic pancreatitis and three had autoimmune pancreatitis. Six patients underwent pancreaticoduodenal resection and four patients, resection of the cauda or cauda and corpus, because malignancy could not be excluded based on preoperative investigations. In two patients the diagnosis of chronic pancreatitis was known before serum sampling. All sera were stored in aliquots of 1 mL at −80°C until used for further processing described below. The study was approved by the Surgical Ethics Committee of Helsinki University Hospital (Dnro HUS 226/E6/06, extension TMK02 §66 17.4.2013). An informed written consent was obtained from all patients.

### Further processing and trypsin digestion

Serum samples were thawed and used for TOP 12 protein depletion using the TOP12 protein depletion kit (Pierce, ThermoFisher) according to the manufacturer's instructions. Total protein concentration was determined in TOP12 protein depleted serum with Pierce BCA assay kit (Pierce, ThermoFisher). Serum equivalent to 100 *μ*g protein was aliquoted and dried by speedvac (Savant, ThermoFisher). The resulting dried serum was dissolved 35 *μ*L in 50 mmol/L Tris buffer, pH 7.8 containing 6M urea. After the protein dissolved, 1.8 *μ*L of 200 mmol/L DTT was added to the samples and allowed to shake at RT for 1 h. Further, 7 *μ*L of iodoacetamide (200 mmol/L stock solution) was added to the samples and they were further allowed to shake at RT for 1 h. After alkylation, to quench excess iodoacetamide and prevent overalkylation 7 *μ*L of DTT (200 mmol/L) was added to samples and again shaken for 1 h at RT. Samples were diluted by adding 270 *μ*L of MQ water and trypsin was added at 1:50 trypsin:protein ratio and digestion was allowed to occur at 37°C overnight. Following the digestion, 30 *μ*g protein equivalent of samples were cleaned with C18 spin columns (Pierce, ThermoFisher). Resulting elution from C18 columns was dried and dissolved in 86 *μ*L of 0.1% formic acid containing 12.5 fmol/*μ*L of Hi3 spike‐in standard peptides (Waters, MA, USA) for quantification.

### Liquid chromatography‐mass spectrometry (LC‐MS) and quantification

#### UPLC‐MS

A quantity of 4 *μ*L samples (equivalent to ~1.4 *μ*g total protein) were injected to nano Acquity UPLC (Ultra Performance Liquid Chromatography) ‐ system (Waters Corporation, MA, USA). TRIZAIC nanoTile 85 *μ*m × 100 mm HSS‐T3u wTRAP was used for separation before mass spectrometer. Samples were loaded, trapped and washed for two minutes with 8.0 *μ*L/min with 1% B. The analytical gradient used is as follows: 0–1 min 1% B, at 2 min 5% B, at 65 min 30% B, at 78 min 50% B, at 80 min 85% B, at 83 min 85% B, at 84 min 1% B and at 90 min 1% B with 450 nL/min. Buffer A: 0.1% formic acid in water and Buffer B: 0.1% formic acid in acetonitrile.

Data were acquired in DIA (data independent acquisition) fashion using HDMSE mode with Synapt G2‐S HDMS (Waters Corporation, MA, USA). The collected data range was 100–2000 m/z, scan time one‐second, IMS wave velocity 650 m/s, collision energy was ramped in trap between 20 and 60 V. Calibration was done with Glu1‐Fibrinopeptide B MS2 fragments and as a lock mass, Glu1‐Fibrinopeptide B precursor ion was used during the runs. The samples were run as triplicates and further analysis was done with, Progenesis QI for Proteomics – software (Nonlinear Dynamics, Newcastle, UK).

#### Data analysis

Data analysis was performed as previously described 7. Briefly, the raw files were imported to Progenesis QI for proteomics software (Nonlinear Dynamics, Newcastle, UK) using lock mass correction with 785.8426 m/z, corresponding to doubly charged Glu1‐Fibrinopeptide B. Default parameters for peak picking and alignment algorithm were used. The software facilitated the peptide identification with Protein Lynx Global Server and label‐free quantification 8. The peptide identification was done against Uniprot human FASTA sequences (UniprotKB Release 2015_09, 20205 sequence entries) with (CLPB_ECOLI (P63285)), ClpB protein sequence inserted for label‐free quantification. Modifications used were as follows: fixed at cysteine (carbamidomethyl) and variable in methionine (oxidation). Trypsin was used as digesting agent and one missed cleavage was allowed. Fragment and peptide error tolerances were set to auto and FDR to less than 4%. One or more ion fragments per peptide, three or more fragments per protein and one or more peptides per protein were required for ion matching. These are default parameters in the software.

The identified proteins are grouped as one according to parsimony principle and also peptides unique to the protein are reported. Parsimony principle states that protein hits are reported as the minimum set that accounts for all observable peptides. Progenesis QI for proteomics does not take a strict parsimonious approach because of over‐stringency as has been pointed out before 9. However, for resolution of conflicts, if two proteins contain some common peptides, protein with fewer peptides is subsumed into the protein with higher number of peptides which are a superset of the subsumed protein's peptides. All relevant proteins are listed as a group under the lead protein with greatest coverage or the highest score when the coverages of two or more proteins are equal. Quantitation is performed using the lead identity peptide data. More details about this approach can be found on the software website (www.nonlinear.com).

The ANOVA calculation assumes that the conditions are independent and applies the statistical test that assumes the means of the conditions are equal. The label‐free protein quantitation was done with Hi‐N method 8. In every injection the sample contained also 50 fmol of six CLPB_ECOLI (P63285, ClpB protein) peptides (Hi3 *E. coli* Standard, Waters). Hi3 peptides are used for normalizing the peptide abundancies and relative quantitation was based on all the non‐conflicting peptides found. The peptide ranking is done across all the runs. The abundancies of the peptides are averaged to provide a signal to the protein. Workings of the Progenesis softwares have been described in details on the software website (www.nonlinear.com) and also in published literature 10. Differences between controls and cases were evaluated with ANOVA on a protein‐to‐protein basis. Principle component analysis was performed with Progenesis QI for proteomics. Analyse‐It program (with Microsoft Excel) was used for calculating area under the curve (AUC) values of ROC curves with all the default parameters.

### Orthogonal partial least square modeling and discriminant analysis (OPLS‐DA)

OPLS‐DA modeling for pancreatitis and pancreatic cancer patients was performed with ropls 11 R package. Quality metrics, variable importance in projection (VIP), permutation diagnostics (1000 random permutations) and detection of outliers were calculated within the rolps package. One predictive component and 1–4 orthogonal components were used. To find out most influential proteins for separation of patient groups, proteins with VIP>1 and univariate *P*‐value < 0.05 adjusted for false discovery rate 12 were selected. As an alternate method to find out most influential proteins, a S‐plot with loadings of each protein on the *X*‐axis and correlation of scores to modeled X‐matrix (p(corr)[1]=Corr(t1,X), t1 = scores in the predictive component) on *Y*‐axis was constructed. Proteins with absolute value of loadings over 0.1 and absolute values of correlations over 0.7 were selected. A sample was considered as a possible outlier, if score distance exceeded SQRT(*χ*2(0.975)). The variables were standardized by mean centering and unit variance scaling. More than two unique peptide and a confidence score of more than 4.5 was required for proteins to be included in the OPLS‐DA model. This filtering based on confidence score is based on previous work 13.

### Pathway analysis

Integrated Molecular Pathway Level Analysis (IMPaLA) was used for pathway over representation analysis. The method and rationale behind the approach has been published previously 14. By default, if no background list is supplied, the software uses all the entities present in all the pathways as background. Ingenuity pathway analysis (Ingenuity Systems, Redwood City, CA) was used for performing core analysis on the proteomic dataset with default parameters of the software. The results (canonical pathways) are presented in the results section as a figure. All the identified proteins were fed into IPA as input.

### Data repository

The raw files were converted with MSConvert (ProteoWizard) to mzML‐files. The mass spectrometry proteomics data have been deposited to the ProteomeXchange Consortium via the PRIDE 15 partner repository with the dataset identifier PXD005144.

## Results

### Metadata

Twenty two pancreatic cancer and 13 chronic pancreatitis samples were analyzed in this study. In the CP group, three patients had autoimmune pancreatitis, and 10 had alcohol‐induced chronic pancreatitis. Age of the patients in the PC group ranged from 54 to 79 and in the CP group from 42 to 74. Complete clinical features of the patients including grade of the tumor, T and N stage, gender and age are given in Table S1. One of the samples (autoimmune pancreatitis) was excluded from the analysis (due to technical reasons) as the chromatographic alignment of the technical replicates was not good and it stacked further aside from other samples in the PCA. This sample is marked in the Table S1.

### Protein identification

We analyzed PC and CP samples by HDMSE and quantified 653 proteins from serum including proteins with minimum one unique peptide. Two hundred and ninety proteins were quantified with two or more unique peptides. Confidence score of identification ranged from 4084.212 to 3.913. Only 11 proteins had the confidence score less than 4.6. Fold changes ranged from 1369.4 to 1.001 when PC has the higher mean and 236.5 to 1.004 when CP had the higher mean. One protein was found only in CP and five only in PC. Hundred and sixty‐four proteins passed the cutoff of 0.05 of ANOVA *P* value when the higher mean was set to CP and 102 when the higher mean was set to PC. The complete data of all the proteins including various parameters such as ANOVA *P* values are given in Table S2. Our main criterion for differing proteins between the two classes was ANOVA *P* values and many proteins have *P* values higher than 0.05 despite having big fold change. Such proteins are not considered to be different.

### Principal component analysis (PCA)

**Figure 1:**
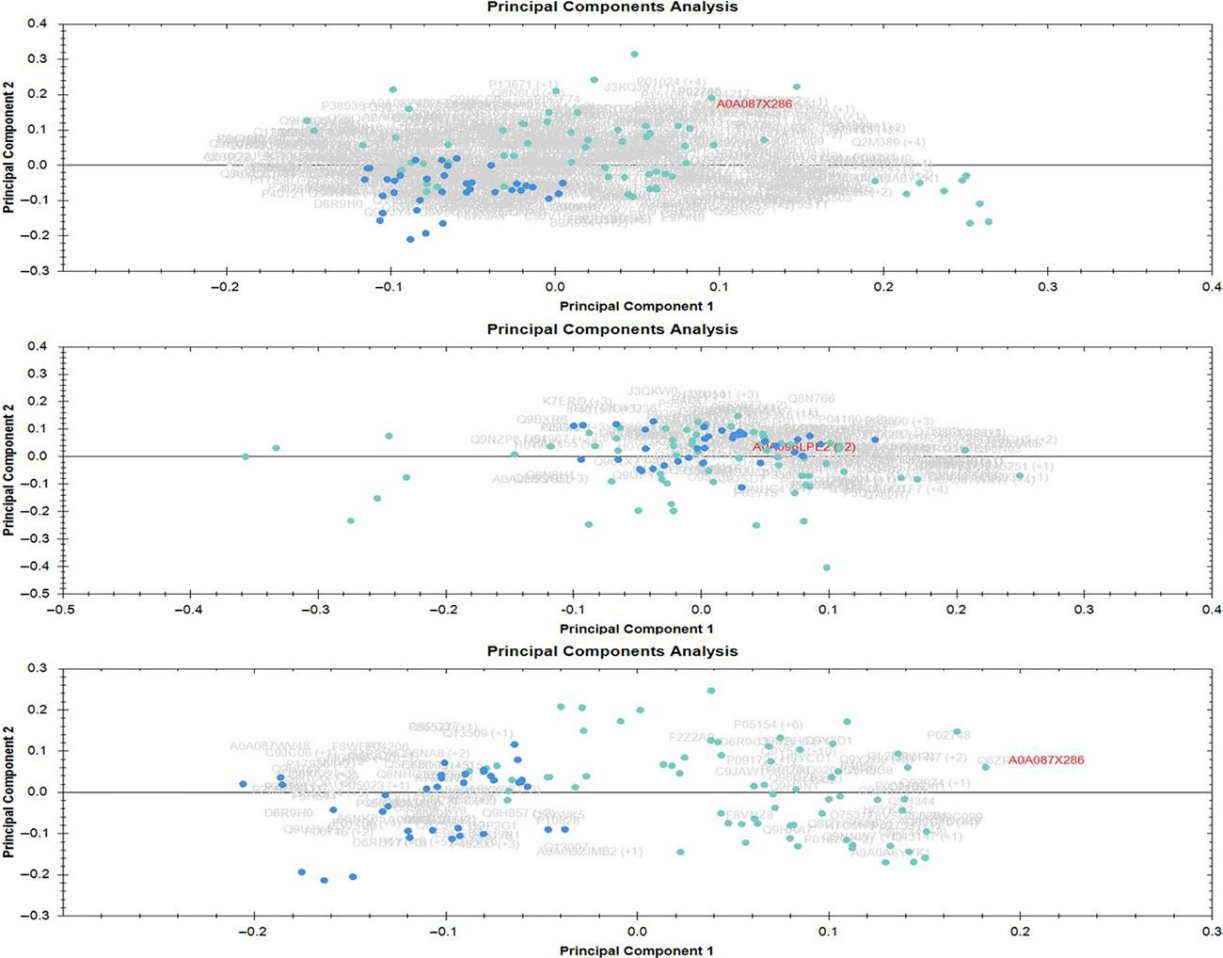
Principal component analysis (PCA). Blue dots are samples of chronic pancreatitis and cyan dots are samples of pancreatic cancer. Each sample was run in triplicates. Upper panel is when the PCA was performed on all proteins with one or more unique peptides and middle panel is when proteins having the fold change of 1.0‐1.3 between the two conditions (housekeeping proteins) were used for PCA. Lower panel depicts the PCA when proteins passing the cutoff of 0.05 for ANOVA were used for PCA.

PCA was performed using the software Progenesis QI for Proteomics. This analysis determines the main axis of variation in the quantities of individual proteins which can point out the outliers. This method is also suitable to study the technical replicates as they should be close to each other on the PCA biplot. PCA biplot can tell the difference between amounts of variation among two classes of samples and present it in a visual manner. PCA of these CP and PC is shown in Figure 1.

The upper panel in the Figure 1 is the PCA when all the proteins were considered for PCA, and it can be seen that samples already have the tendency to cluster into two classes. Some overlapping samples can also be seen between the two groups but majority of the samples from the two groups (CP and PC) fall apart on the biplot. When only the housekeeping proteins (fold change 1 to 1.3) were considered for PCA (middle panel) there is an almost complete overlap between the two groups which is expected as these proteins do not differ much between the two classes. Further, when only the proteins, having ANOVA *P* values lower than 0.05 and fold change more than 2, were considered for PCA there is a clear separation of the two groups with only very few samples falling in the overlapping area. The better than moderate separation of PC and CP patients in PCA prompted us to delve deeper into the statistical analysis and we constructed a model which is described in the next section.

### Orthogonal partial least square modeling and discriminant analysis (OPLS‐DA)

OPLS method enables distinct modeling of the predictive or correlated variance to the factor of interest as well as the uncorrelated variance. OPLS is an extension of the PLS statistical modeling but performs better in terms of interpreting the data from the model 11. It is particularly useful when the number of variables exceeds the number of samples and when there is multicollinearity among the variables. For constructing an OPLS‐DA model, *ropls* package in “R” was used and as a first step the data was filtered. For the filtering of proteins, stringent parameters were used such as; only the proteins with more than two peptide count and more than two unique peptides with confidence score cutoff of 4.5 were considered for OPLS‐DA modeling. These proteins are given in Table S3.

**Figure 2:**
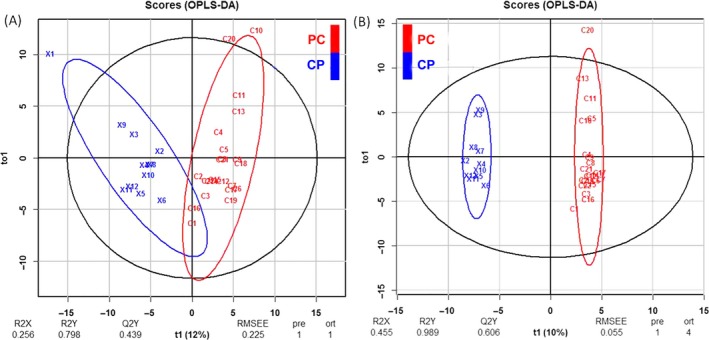
Multivariate modeling of pancreatic cancer (PC) and chronic pancreatitis (CP) patients using OPLS‐DA. A. Score plot of model including all patients (the predictive component on the *x*‐axis and the orthogonal component on the *y*‐axis) B. Score plot of the model excluding two outlier patients (these patients are indicated in the Table S1). R2X and R2Y are proportion of predictor/response variation explained by the full model, respectively. Q2Y is predictive performance of the model, RMSEE is root mean squared error of estimation, pre is the number of predictive components, ort is number of orthogonal components.

Multivariate model was built, using OPLS in *ropls* package, for each protein's abundance. The scores plot for the model is shown in the Figure 2A. The model was built with one predictive and one orthogonal component. Black ellipse is the 95% confidence interval for the model and red and blue ellipses are the 95% of multivariate normal distribution for each class of samples.

Permutation testing was employed to establish the significance of the R2Y and Q2Y values (Figure S1) and *P* values for both parameters can be found on the top of the graph (both being significant). Diagnostic plot (Figure S2) was used to highlight the observations to find out the samples which are further away from the projection plane.

Cutoff for the score distance is the vertical dotted line and patient numbered C10 and ×1 appear to be outliers (Figure 2A). These two samples are marked in the Table S1 in the remarks column, which is patient information table. To further assess whether the quality of the model (or fit, R2Y) or separation between the classes (Q2Y) can be improved by removing the outliers, these two samples were removed and model was built again. After removing these two outliers, the model was built again and this time we called it improved model. Figure 2B shows the scores plot and the permutation testing is shown in Figure S1 of the rebuilt, Improved model.

For cross‐validation, 80% of the data was used for the training of the model and 20% for the prediction, averaging over 1000 randomly chosen training and prediction sets. We calculated the sensitivity and specificity of the prediction in the improved model using this approach and it was found to be 94% (95% CI: 93–95%) and 67% (95% CI: 65–68%), respectively. In the training series or in the full model sensitivity and specificity were both 100%.

**Figure 3:**
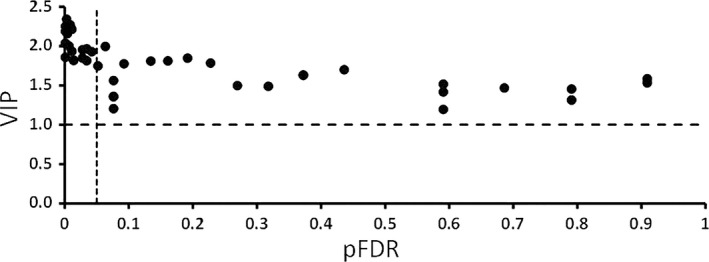
Selection of influential proteins. Proteins which had variable importance in projection (VIP) >1 and *P* ‐values adjusted for multiple comparisons <0.05 were considered as influential proteins for the separation of pancreatic cancer and chronic pancreatitis patients. The vertical dotted line is cutoff 0.05 for pFDR and horizontal dotted line is the cutoff 1 for VIP.

**Figure 4:**
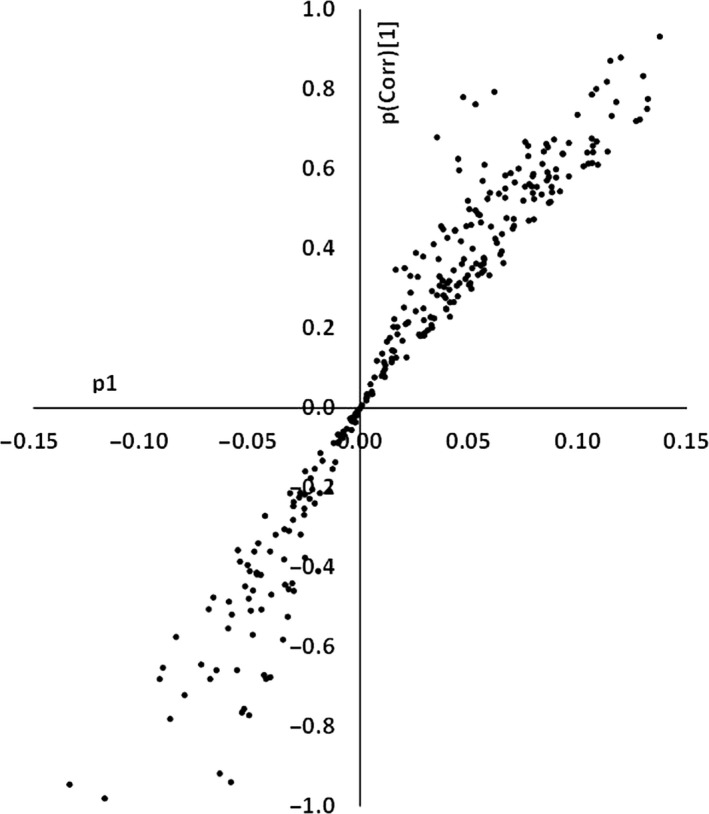
Selection of influential proteins, S‐plot Protein loadings (p1) on the *X*‐axis and p(Corr)[1] on *Y*‐axis. Proteins are coded by numbers. Loading vector was on the *x*‐axis and correlation score on *y*‐axis. An absolute cutoff value of the 0.1 for loading score and an absolute value of 0.7 for the correlation score was used to filter the significant proteins.

To find out the proteins of interest, which were significant sources of variation between the two classes, two approaches were taken (univariate vs. multivariate parameters plot and S‐Plot). *P* value for the false discovery rate (pFDR, univariate parameter) and variable influence on projection (VIP, multivariate parameter) were calculated for all the proteins and plotted against each other (Fig. 3). To validate this list of significantly different proteins and to find the variable proteins by another method S‐Plot was constructed (Fig. 4).

Nineteen significant proteins were found by pFDR versus the VIP plot in the initial model and 18 in the improved model. Fifteen proteins were found to be significant in the improved model S‐Plot (Table 1). Most proteins were common in all three methods and can be seen in the Table 1.

**Table 1:** Selection of significantly different proteins

Initial model VIP vs. pFDR				Improved model VIP vs. pFDR				Improved model S‐Plot			
Uniprot Accessions	Protein Name	*P*FDR	VIP	Uniprot Accessions	Protein Name	*P*FDR	VIP	Uniprot Accessions	Protein Name	*P*1	*P*(corr)[1]
P02760;S4R471	Protein AMBP	0.0003	2.1372	P04217	Alpha‐1B‐glycoprotein	0.0008	1.8576	Q8IVV2;H7BZ41;J3QKX9	Lipoxygenase homology domain‐containing protein 1	−0.1170	−0.9794
P19823;A0A087WTE1;Q5T985	Inter‐alpha‐trypsin inhibitor heavy chain H2	0.0005	1.9313	P00734;C9JV37;E9PIT3	Prothrombin	0.0008	2.0369	P26927;H7C0F8	Hepatocyte growth factor‐like protein	−0.1330	−0.9445
P15313;C9JL73;C9JZ02	V‐type proton ATPase subunit B, kidney isoform	0.0009	1.9485	P02760;S4R471	Protein AMBP	0.0012	2.1869	P15313;C9JL73;C9JZ02	V‐type proton ATPase subunit B, kidney isoform	0.1370	0.9313
P26927;H7C0F8	Hepatocyte growth factor‐like protein	0.0021	2.2957	P19823;A0A087WTE1;Q5T985	Inter‐alpha‐trypsin inhibitor heavy chain H2	0.0012	2.2491	P00734;C9JV37;E9PIT3	Prothrombin	0.1200	0.8786
P36955;I3L107;I3L1U4;I3L2R7;I3L3Z3;I3L425;I3L4F9;I3L4N7;I3L4Z0	Pigment epithelium‐derived factor	0.0021	1.7997	P15313;C9JL73;C9JZ02	V‐type proton ATPase subunit B, kidney isoform	0.0029	2.3420	P05156;D6R9Z8;E7ETH0;G3XAM2	Complement factor I	0.1150	0.8704
P04217	Alpha‐1B‐glycoprotein	0.0034	1.5699	P02790;Q9BS19;Q9NPA0	Hemopexin	0.0039	2.1569	P02743	Serum amyloid P‐component	0.1300	0.8326
P00734;C9JV37;E9PIT3	Prothrombin	0.0043	1.6451	P36955;I3L107;I3L1U4;I3L2R7;I3L3Z3;I3L425;I3L4F9;I3L4N7;I3L4Z0	Pigment epithelium‐derived factor	0.0067	2.0018	P43652	Afamin	0.1130	0.8184
P19827;F8WAS2;H7C0N0;H7C5I0	Inter‐alpha‐trypsin inhibitor heavy chain H1	0.0043	1.9809	P26927;H7C0F8	Hepatocyte growth factor‐like protein	0.0086	2.2709	P01031	Complement C5	0.1080	0.8001
P01024;E9PJV1;E9PR27;M0QYC8;O95568	Complement C3	0.0069	1.6420	P19827;F8WAS2;H7C0N0;H7C5I0	Inter‐alpha‐trypsin inhibitor heavy chain H1	0.0086	2.2428	Q9Y5I0;C9JA99;D6RA20;Q9UN74;Q9UN75;Q9Y5H5;Q9Y5H7;Q9Y5H8;Q9Y5H9;Q9Y5I3;Q9Y5I4	Protocadherin alpha‐13	0.1060	0.7860
P05155;H0YCA1;H9KV48	Plasma protease C1 inhibitor	0.0086	1.9650	P02743	Serum amyloid P‐component	0.0110	2.2131	P19823;A0A087WTE1;Q5T985	Inter‐alpha‐trypsin inhibitor heavy chain H2	0.1320	0.7748
P05156;D6R9Z8;E7ETH0;G3XAM2	Complement factor I	0.0107	1.7204	P01024;E9PJV1;E9PR27;M0QYC8;O95568	Complement C3	0.0110	1.9340	P36955;I3L107;I3L1U4;I3L2R7;I3L3Z3;I3L425;I3L4F9;I3L4N7;I3L4Z0	Pigment epithelium‐derived factor	0.1170	0.7672
Q8IVV2;H7BZ41;J3QKX9	Lipoxygenase homology domain‐containing protein 1	0.0161	1.7367	E9PG39;E9PC15;Q53H12	Acylglycerol kinase, mitochondrial	0.0139	1.8189	P19827;F8WAS2;H7C0N0;H7C5I0	Inter‐alpha‐trypsin inhibitor heavy chain H1	0.1320	0.7499
Q6ZRR7;H3BUS4	Leucine‐rich repeat‐containing protein 9	0.0161	1.7785	Q06033;A0A087WW43;E7ET33	Inter‐alpha‐trypsin inhibitor heavy chain H3	0.0139	1.8199	P02749;J3KS17;J3QLI0;J3QRN2	Beta‐2‐glycoprotein 1	0.1150	0.7322
P43652	Afamin	0.0161	1.8528	Q6ZRR7;H3BUS4	Leucine‐rich repeat‐containing protein 9	0.0276	1.8487	P02760;S4R471	Protein AMBP	0.1280	0.7236
P02790;Q9BS19;Q9NPA0	Hemopexin	0.0241	1.8158	P05156;D6R9Z8;E7ETH0;G3XAM2	Complement factor I	0.0276	1.9549	P02790;Q9BS19;Q9NPA0	Hemopexin	0.1270	0.7193
Q9C099	Leucine‐rich repeat and coiled‐coil domain‐containing protein 1	0.0291	1.3278	P02749;J3KS17;J3QLI0;J3QRN2	Beta‐2‐glycoprotein 1	0.0341	1.9661				
P02743	Serum amyloid P‐component	0.0291	1.6691	P05155;H0YCA1;H9KV48	Plasma protease C1 inhibitor	0.0341	1.8136				
P02749;J3KS17;J3QLI0;J3QRN2	Beta‐2‐glycoprotein 1	0.0291	1.7742	P43652	Afamin	0.0422	1.9287				
E9PG39;E9PC15;Q53H12	Acylglycerol kinase, mitochondrial	0.0424	1.5528								

The OPLS‐DA model was built initially and again after removing two outliers found in the initial model. Variable influence on projection (VIP) values were plotted against *P* value for the false discovery rate (pFDR) and significantly different proteins between the two disease conditions were selected by choosing a cutoff of 0.05 for pFDR and 1 for VIP. These proteins as well as the proteins found to be significantly different by S‐Plot (*P* 1 cutoff value of 0.1 and *P* (Corr) [1] value of 0.7) are presented in the table with appropriate parametric values given for each protein.

### Pathway analysis

Two tools were employed for pathway analysis namely IMPaLA and Ingenuity pathway analysis (IPA). Figure S3 shows the pathway over‐representation analysis by IMPaLA in two conditions; when the highest mean was set to CP and when the highest mean was set to PC. Only the pathways with P_genes and Q_genes values below 0.05 were considered and Figure S3 shows the top 10 pathway enriched in both the lists of proteins. Various complement activation and coagulation pathways were the main pathways enriched in both the lists. However, P_genes and Q_genes values were better in PC list for these pathways compared to CP list. Fibrinolytic pathways were particular enriched in CP and not in PC list. In IPA core analysis, top biofunctions and diseases are shown in Figure S4. Inflammatory response, developmental disorder and immunological diseases were the top disorders enriched and cellular movement and lipid metabolism were among the top molecular functions enriched.

**Figure 5:**
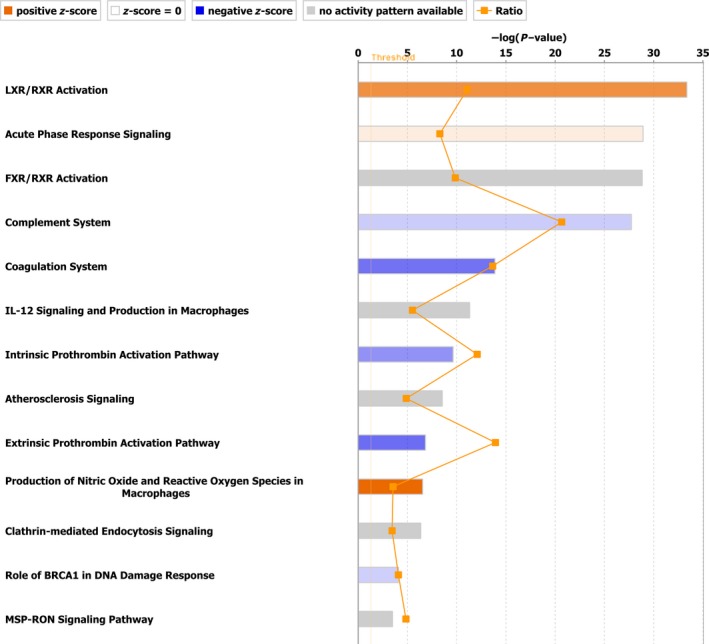
Canonical pathways enriched by core analysis in IPA. Top canonical pathways enriched by Ingenuity Pathway Analysis “Core analysis” are shown here. Straight orange vertical line running through the bars is threshold for *P* value for the particular pathway's enrichment. Horizontal axis is the –log (*P* value) and vertical axis represents the given pathways.

In the same analysis by IPA, multiple canonical pathways were enriched in the CP and PC proteomic dataset. These were LXR/RXR activation, acute phase signaling response and similar to IMPaLA analysis, complement and coagulation system. Some of the top canonical pathways are shown in Figure 5.

### Receiver operating characteristic (ROC) curves

Proteins found most significantly different in initial and improved OPLS‐DA model and S‐Plot of OPLS‐DA model were further used for ROC Curve analysis. This method was utilized to validate the diagnostic value of the proteins found common to all three methods, results of which are presented in Table 1. All these proteins were compared with each other to get a list of unique proteins and common to all three techniques. These proteins were analyzed by ROC curve analysis and area under the curve (AUC), 95% confidence interval and standard error were calculated as described in methods. The results are presented in Table 2.

**Table 2:** Receiver operating characteristics curve analysis of the proteins found to be significantly different in PC vs. CP OPLS‐DA analysis and pFDR vs. VIP analysis

Protein Name	AUC	Lower	Upper	*P*	Sensitivity (%)	Specificity (%)
Alpha‐1B‐glycoprotein	0.920	0.827	1.000	0.000	82	92
Prothrombin	0.917	0.818	1.000	0.000	86	83
Protein AMBP	0.951	0.863	1.000	0.000	91	100
Inter‐alpha‐trypsin inhibitor heavy chain H2	0.947	0.871	1.000	0.000	91	100
V‐type proton ATPase subunit B, kidney isoform	0.939	0.847	1.000	0.000	91	100
Hemopexin	0.886	0.758	1.000	0.000	86	92
Pigment epithelium‐derived factor	0.928	0.837	1.000	0.000	86	92
Hepatocyte growth factor‐like protein	0.928	1.000	0.828	0.000	100	83
Inter‐alpha‐trypsin inhibitor heavy chain H1	0.917	0.825	1.000	0.000	77	100
Serum amyloid P‐component	0.883	0.761	1.000	0.000	95	75
Complement C3	0.909	0.805	1.000	0.000	82	92
Acylglycerol kinase, mitochondrial	0.875	0.756	0.994	0.000	82	83
Inter‐alpha‐trypsin inhibitor heavy chain H3	0.848	0.690	1.000	0.001	86	83
Leucine‐rich repeat‐containing protein 9	0.894	0.788	1.000	0.000	77	100
Complement factor I	0.902	0.797	1.000	0.000	91	83
Beta‐2‐glycoprotein 1	0.883	0.772	0.993	0.000	68	100
Plasma protease C1 inhibitor	0.905	0.800	1.000	0.000	86	92
Afamin	0.894	0.777	1.000	0.000	86	92
Lipoxygenase homology domain‐containing protein 1	0.894	1.000	0.788	0.000	95	67
Protocadherin alpha‐13	0.841	0.703	0.978	0.001	86	83

Area under the curve values (AUC), lower and upper confidence values, *P* value, sensitivity and specificity are given in the table.

The Highest AUC value was found for protein AMBP (AUC: 0.951) followed by Inter‐alpha‐trypsin inhibitor heavy chain H2 (AUC: 0.947). The lowest AUC for the proteins analyzed by ROC curve was for protocadherin alpha‐13, which was 0.841 which is also good value for AUC. These proteins can classify the samples into two categories by various methods.

## Discussion

Pancreatic cancer is the eighth leading cause of death worldwide in men and ninth in women 16. The incidence rates are slightly higher in western industrialized world and lower in developing countries such as India and Nigeria. Cancer statistics have revealed that prognosis of pancreatic cancer has not improved in recent years (judged by the survival of the patients) compared to other carcinomas 17. Late diagnosis and aggressive nature of the cancer are responsible for the poor prognosis of PC. Delay in diagnosis is due to the several reasons including the prominent lack of suitable screening markers in an asymptomatic population.

CA 19‐9 and carcinoembryonic antigen (CEA) are the two most used serum biomarkers in PDAC, but are not suitable for screening an asymptomatic population 18, 19. They further have inadequate accuracy, even for primary diagnosis of symptomatic patients 18, 19. Low sensitivity of the test necessitates invasive confirmatory examinations to confirm diagnosis. CA 19‐9 displays sensitivity of 68% at 37 U/mL 1 and CEA 45%^20^ and specificity of 70% and 81%, respectively 1, 20, figures that are too low for differential diagnosis from CP. It has to be noted that different studies report different values of sensitivity for CA 19‐9 due to the differential cutoff values used. CA19‐9 is now mainly accepted only as a follow‐up and prognostic biomarker. Different types of CP cases closely resemble PC in clinical and imaging features and there is urgent need for novel biomarkers for differential and early diagnosis.

Serum is attractive for discovering screening and other type of biomarkers as it reflects organ‐originated physiological changes 21 and is easy to collect. Dynamic range of protein concentration in plasma or serum is 10^9^ 22 and top 12 proteins constitute >95% of total plasma/serum proteins. For this reason, it is very difficult to identify the low‐abundant proteins which are potential biomarkers of various diseases. It is desirable to deplete high‐abundant proteins to enable relatively deeper analysis of the serum proteome in health and disease. Twelve CP and 22 PC patients were retrospectively recruited for this study and serum protein profiles were studied by HDMS^E^ using Synapt G2S HDMS system. After depletion of the top 12 most abundant proteins in human serum as described in methods, we quantified 291 proteins. However, Out of the total proteins identified, only some proteins (with peptide count of 2 or more and 2 or more unique peptides with confidence score of at least 4.5) were used for OPLS‐DA modeling. R package *ropls* was employed to this end and data was normalized as described in methods. PCA visually presents the principal axes of variation in samples which helps in interpreting the separation of the groups. However, PCA with outliers present can give false results or lead to incorrect interpretations in classification of the samples. OPLS‐DA scores plot and diagnostic plot can easily spot outliers in the data. These outliers, if included in the modeling reduce the optimum fit of the model to the data and lower the predictive accuracy of the statistical model. We found two outliers in the data and they were removed and the model was built again. This exercise markedly improved the separation quality of the model. These outliers performed normally as technical replicates however, the standardized expression profile for one of these samples (C10) was the highest in its group (Pancreatic cancer) and the other sample (×1) had the lowest expression profile in its group (Chronic pancreatitis). It could have been the reason for them to perform differently from the rest of the samples. Proteins of interest were found in the initial model by pFDR versus the VIP plot (univariate) and in improved model by univariate as well as multivariate manner (S‐Plot). Most of the proteins found were common to all three lists suggesting that these proteins are the main sources of variation among the two disease groups. It also suggests that these two methods are complementary to each other for feature selection in OMICS data which is in accordance with published literature 11.

Acylglycerol kinase, mitochondrial (AGK), is a lipid kinase which converts mono‐and diacylglycerol to lysophosphatidic acid (LPA) and phosphatidic acid (PA) respectively 23. Increase in LPA results in transactivation of epidermal growth factor receptor (EGFR) which leads to increased cell proliferation 23. Overexpression of AGK can drive cancer cell growth and play an important role in pathophysiology of cancer 23. Protein AMBP was another protein which was commonly found in all two models and all three methods of feature selection to be significantly different (Table 1). It has previously been found to be increased in pancreatic cancer 24. However, there is no study hinting at its pathophysiological role in PC or CP. AMBP is an acute phase protein like others found in our study to be significantly different between PC and CP such as afamin, hemopexin and inter alpha‐trypsin inhibitor heavy chain (ITIH) H1, H2, and H3. Acute phase response (APR) is critical for our bodies to respond to injury. However, sustained APR can lead to development of chronic inflammation and tissue injury eventually giving rise to diseases such as cancer. Acute phase proteins also increase the blood flow to the site of injury 25 which can be hijacked by tumor microenvironment as a strategy to get access to sustained blood flow and nutrients. Therefore, acute phase proteins such as those found in our study are not bystanders but potentially active players in tumorigenesis. They can make suitable biomarkers but it has to be considered with caution as they go up in several other conditions as well 26, 27. Acute phase proteins may also reflect systemic inflammatory response seen in some of the cancer patients. For example, preoperative systemic inflammatory response (elevated CRP) in PDAC is an indicator of poor prognosis 28. Preoperative CRP levels are also strong predictors of survival in colon cancer 29 and colorectal liver metastases 30. Another protein found in all three methods of feature selection was Hepatocyte growth factor which is a ligand for c‐MET. Cells in tumor microenvironment overexpress HGF and cancer cells have increased expression of c‐MET and these events lead to promotion of various cancer‐driving pathways 31, 32. Our study gives insight into the biology of events driving the sustained growth and invasion of pancreatic cancer. Differential proteomics combined with statistical/mathematical analysis can provide biomarker candidates and also targets for therapeutic modulation of diseases. Two of the proteins, protein AMBP and ITI‐H2 are known to be modified by chondroitin sulfate 33 and linked to each other. In protein AMBP, we have identified peptides which belong to only the Trypstatin part of the protein. Trypstatin is a separate chain which is a monomer. We did not identify any peptide from other parts of the protein AMBP (Bikunin) which, considering the limitations of the technological workflow used, excludes the possibility that we quantified the complex of ITIH2 heavy and light chain. In case of ITIH2, we have identified peptides only from the mature part of the chain while the chondroitin sulfated peptide is in propeptide region from which we did not identify any peptide. This again, despite the limitations, excludes the possibility that we have quantified the ITIH2 complex with light chain bound via chondroitin sulfate.

In the context of published literature, the study by Pan et al. quantified four plasma biomarkers of PC namely, 14‐3‐3 sigma, gelsolin, lumican and tissue inhibitor of metalloproteinase 1 (TIMP‐1)^34^. When compared to our dataset, 14‐3‐3 sigma and TIMP‐1 were not identified in our study. The reason is the low concentration of these two proteins (approximately 50 ng/mL for 14‐3‐3 sigma and 700 nag/mL for TTIMP‐1). The other two proteins gelsolin and lumican were confidently identified in our study. In the study by Pan et al. gelsolin had a fold change (FC) of 3.4 in PC versus CP and our study has the FC of 1.3 in PC vs CP. It was significantly different in both the studies (T‐test in Pan et al. and Mann–Whitney test in our study). However, Lumican, having the FC of 1.18 in our study (PC vs. CP) and 2.62 in Pan et al. (PC vs. CP) was not found to be significantly different in our study by Mann–Whitney test. It is to be noted that it was significantly different in our study by T‐test but not by Mann–Whitney test. Pan et al. did the T‐test for finding significance among the differences. Non‐normal distribution dictates the use of non‐parametric test and because of this reason, Mann–Whitney test was used in our study. Careful choice of statistical tests allows for more robust data analysis and chance of finding real differences are increased. However, none of these proteins passed the significance in OPLS‐DA modeling as being significantly different proteins. T‐test is a univariate method of data analysis while modern day proteomics datasets are more amenable to multivariate data analysis such as OPLS‐DA modeling. Out of the 653 proteins identified in our study, 257 are previously known to be present in plasma/serum while 396 proteins were identified for the first time. In one of the tissue proteomics study on PDAC tissue samples, 525 proteins were identified and 23 of them were common to our study including gelsolin, complement C3 and lumican 35. However, from one mouse study on PDAC tissues, the results did not overlap much 36. In one study in pancreatic juice from CP and PC, some of proteins found to be differentially expressed also overlapped with our study from serum 37. These proteins were hemopexin, Beta‐2‐glycoprotein 1, Alpha‐1B‐glycoprotein and complement C3. Hemopexin as a glycoprotein biomarker from serum samples was also found to be able to classify CP versus PC samples in another study 38. Another study identifying fucosylated proteins as candidate biomarkers for PC versus CP classification found 14 proteins common with our study 39. These proteins are Plasma protease C1 inhibitor, hemopexin, Alpha‐1B‐glycoprotein, Inter‐alpha‐trypsin inhibitor heavy chain H2 and H1, Complement C5, Serum amyloid P‐component, Complement factor I, Protein AMBP, Beta‐2‐glycoprotein 1, Prothrombin, Pigment epithelium‐derived factor, Afamin and Complement C3. Other proteins suggested as candidate biomarkers in our study such as Hepatocyte growth factor‐like protein and Acylglycerol kinase, mitochondrial among others are novel and presented for the first time to the best of our knowledge.

Pathway analysis mainly yielded the complement and coagulation cascades as enriched suggesting that they are the main perturbed pathways. Tissue factor, plasminogen and thrombin are the main coagulation‐related proteins increased in PC 40, 41, 42. Such a situation will lead to hypercoagulability‐like state which is frequently observed in PC. It has been previously shown that thrombin:antithrombin complex and prothrombin fragment 1 + 2 are increased in CP compared to healthy individuals but they are lower than in PC 43. We have found, in our dataset, that prothrombin is increased in PC compared to CP with significant ANOVA *P* ‐value. In the OPLS‐DA analysis the *P* value was also found to be significant for this protein (*P* = 0.00087 i.e., *P* < 0.001, see Table 1). Moreover, the prothrombin:antithrmobin ratio in our dataset in was 0.29 in CP while it was 0.34 in PC which, in the form of a trend, agrees well with the literature 43. Our pathway analysis results show that *P* and Q values for coagulation pathways were much stronger for PC compared to CP. It could be a continuous phenomenon and with increasing inflammation the thrombin levels are increased and become highest for PC. In such a case, an optimum cutoff value can differentiate PC from CP. Accordingly, AUC for thrombin was found to be 0.917 which is one of the highest in our dataset.

In conclusion, we report a number of potential biomarkers with good statistical significance which can be used to differentiate PC versus CP. This is one of the most cumbersome and difficult clinical decisions in certain age groups. This pilot research paves the way for further studies to capitalize on the potential of the current one.

## Conflict of Interest

Authors declare no conflict of interest.

## Supporting information


**Figure S1.** Permutation testing of significance of R2Y and Q2Y values. A. Model including all patients, B. Model excluding two suspected outlier patients. 1000 permutations were performed.
**Figure S2.** Score distance versus the orthogonal distance plot for the dataset. The score distance cutoff line is the vertical dotted line, the orthogonal distance cutoff line is the horizontal dotted line. Any sample lying to the right of the vertical line or to the top of the horizontal line can be considered as outlier.
**Figure S3.** Pathway over‐representation analysis by IMPaLA. Pathway enrichment analysis using IMPaLA web based server was performed on two proteins list, one having highest mean in chronic pancreatitis (CP, left panel) and other having highest mean in pancreatic cancer (PC, right panel). P‐value is given in blue bars while Q values are represented by red bars.
**Figure S4.** Molecular and cellular functions in IPA core analysis. Ingenuity pathway analysis “core analysis” was performed on the proteomic dataset and top molecular and cellular functions and disease and disorders are given here.

**Table S1**. Pancreatic cancer samples: All patients were M0, had no distant metastases.

**Table S2**. All the proteins quantified in the study with one or more unique peptides. Accession, peptide count (total peptides) and unique peptides, confidence score for identification, ANOVA p values for each protein, maximum fold change and the highest and lowest mean conditions are given in the table along with the full protein name in the description heading.

**Table S3**. Proteins included in OPLS‐DA modeling post‐filtering based on two unique peptides and confidence score of more than 4.5.
