# Supplementary material for: Comparative proteomic profiling of the serum differentiates pancreatic cancer from chronic pancreatitis
Source: Cancer Med. 2017 Jun 1;6(7):1738–51. doi: 10.1002/cam4.1107 (PMC5504330; doi:10.1002/cam4.1107)
Supplement: Supplementary file 1 — Figure S1. Permutation testing of significance of R2Y and Q2Y values. A. Model including all patients, B. Model excluding two suspected outlier patients. 1000 permutations were performed. Figure S2. Score distance versus the orthogonal distance plot for the dataset. The score distance cutoff line is the vertical dotted line, the orthogonal distance cutoff line is the horizontal dotted line. Any sample lying to the right of the vertical line or to the top of the horizontal line can be considered as outlier. Figure S3. Pathway over‐representation analysis by IMPaLA. Pathway enrichment analysis using IMPaLA web based server was performed on two proteins list, one having highest mean in chronic pancreatitis (CP, left panel) and other having highest mean in pancreatic cancer (PC, right panel). P‐value is given in blue bars while Q values are represented by red bars. Figure S4. Molecular and cellular functions in IPA core analysis. Ingenuity pathway analysis “core analysis” was performed on the proteomic dataset and top molecular and cellular functions and disease and disorders are given here. [file CAM4-6-1738-s001.doc]

**Comparative proteomic profiling of the serum differentiates pancreatic cancer from chronic pancreatitis**


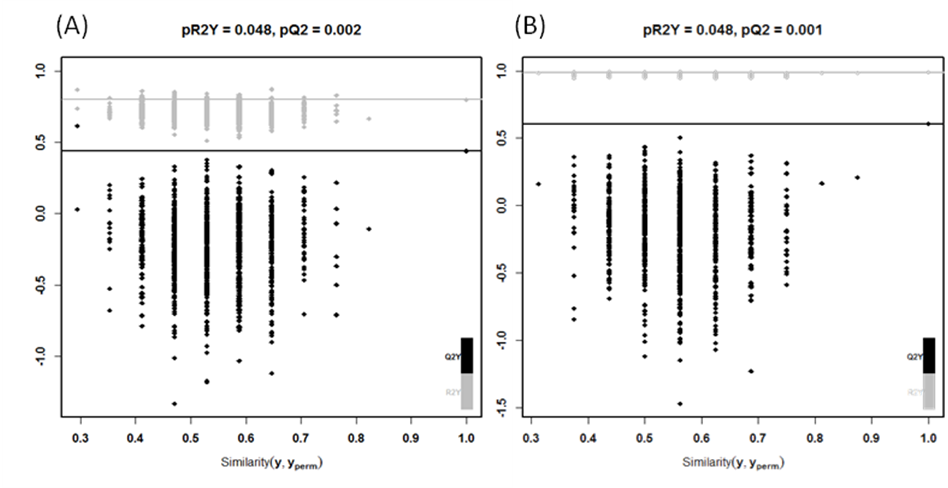


**Supplementary Figure S1:** **Permutation testing of significance of R2Y and Q2Y values.** A. Model including all patients, B. Model excluding two suspected outlier patients. 1000 permutations were performed.


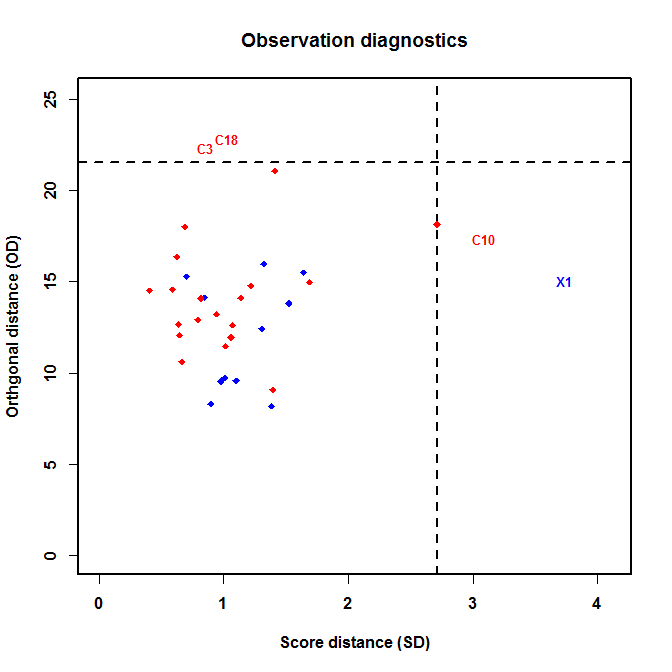


**Supplementary Figure S2: Score distance versus the orthogonal distance plot for the dataset.** The score distance cutoff line is the vertical dotted line, the orthogonal distance cutoff line is the horizontal dotted line. Any sample lying to the right of the vertical line or to the top of the horizontal line can be considered as outlier.


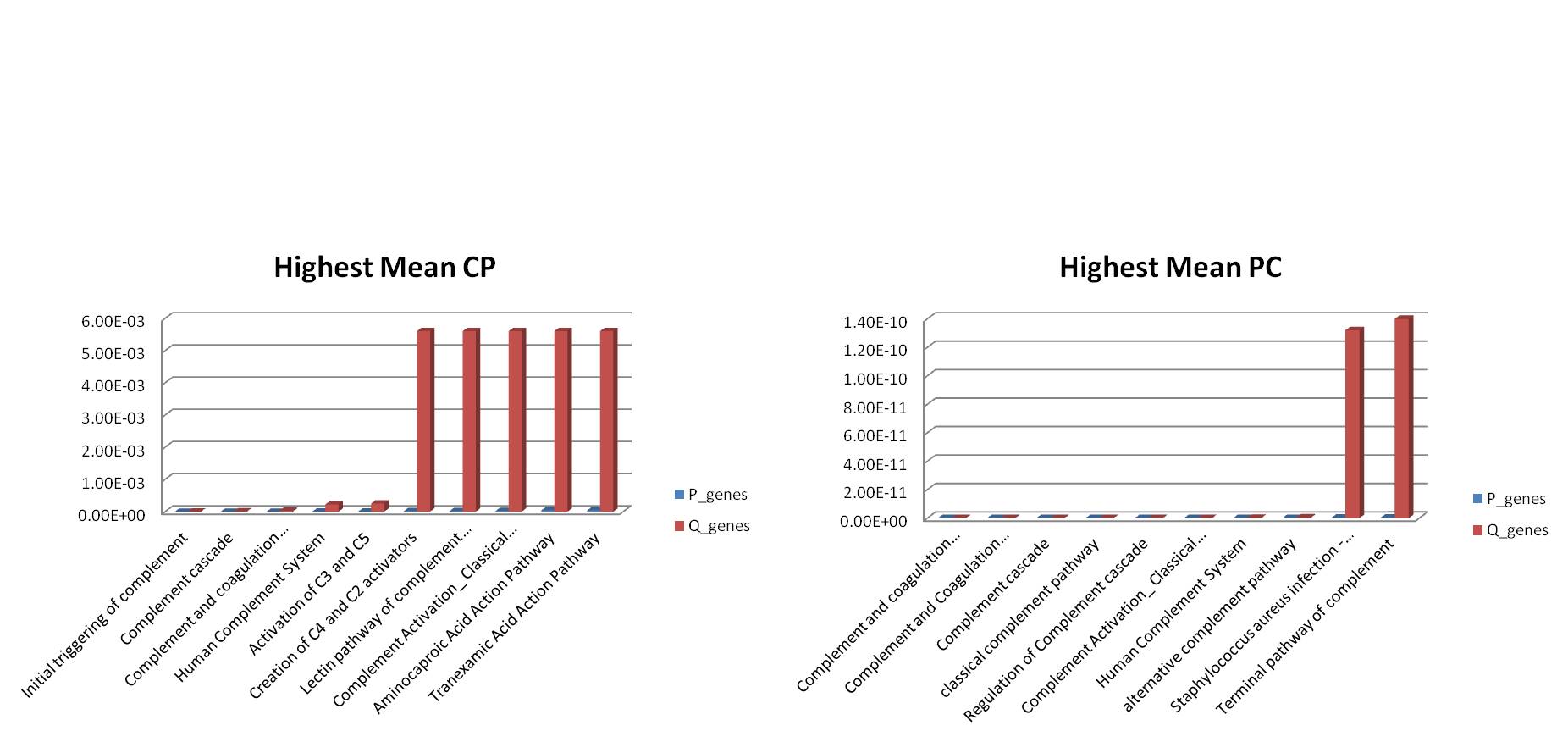


**Supplementary Figure S3: Pathway over-representation analysis by IMPaLA.** Pathway enrichment analysis using IMPaLA web based server was performed on two proteins list, one having highest mean in chronic pancreatitis (CP, left panel) and other having highest mean in pancreatic cancer (PC, right panel). P-value are given in blue bars while Q values are represented by red bars.


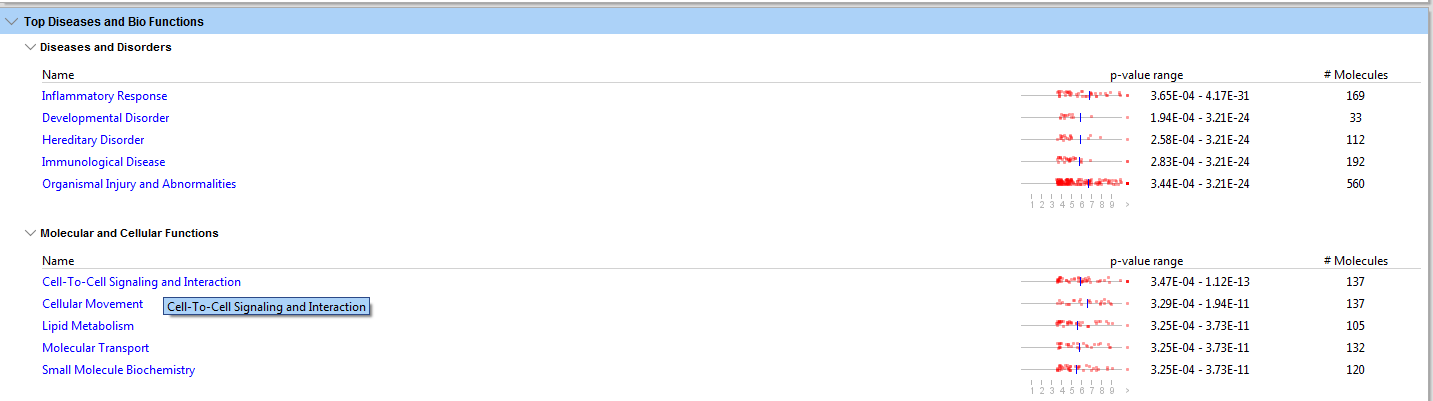


**Supplementary Figure S4: Molecular and cellular functions in IPA core analysis.** Ingenuity pathway analysis “core analysis” was performed on the proteomic dataset and top molecular and cellular functions and disease and disorders are given here.
